# Hallmarks of glycosylation in cancer

**DOI:** 10.18632/oncotarget.8155

**Published:** 2016-03-17

**Authors:** Jennifer Munkley, David J. Elliott

**Affiliations:** ^1^ Institute of Genetic Medicine, Newcastle University, Newcastle-upon-Tyne, NE1 3BZ, UK

**Keywords:** cancer, glycosylation, hallmarks, glycans, aberrant

## Abstract

Aberrant glycosylation plays a fundamental role in key pathological steps of tumour development and progression. Glycans have roles in cancer cell signalling, tumour cell dissociation and invasion, cell-matrix interactions, angiogenesis, metastasis and immune modulation. Aberrant glycosylation is often cited as a ‘hallmark of cancer’ but is notably absent from both the original hallmarks of cancer and from the next generation of emerging hallmarks. This review discusses how glycosylation is clearly an enabling characteristic that is causally associated with the acquisition of all the hallmark capabilities. Rather than aberrant glycosylation being itself a hallmark of cancer, another perspective is that glycans play a role in every recognised cancer hallmark.

## INTRODUCTION

The hallmarks of cancer were originally outlined in 2000 and comprise six biological capabilities acquired during the multi-step development of cancer that allow cancer cells to survive, proliferate and disseminate [1]. As cells evolve progressively to a neoplastic state they acquire a succession of these hallmarks to allow them to become tumourigenic and ultimately malignant. The hallmarks include: sustaining proliferative signalling, evading growth suppressors, resisting cell death, enabling replicative immortality, inducing angiogenesis, and activating invasion and metastasis [1]. Underlying theses hallmarks are genome instability and inflammation which contribute to multiple hallmark functions [1, 2]. In 2011, more than a decade after the publication of the original cancer hallmarks paper, the next generation of cancer hallmarks were published, and two emerging hallmarks were proposed: reprogramming of energy metabolism and evading immune destruction [3]. The next generation of cancer hallmark traits recognised the ‘tumour microenvironment’, or the cellular environment in which the tumour exists, as contributing to the acquisition of hallmark traits, adding another dimension of complexity to cancer progression [3].

Aberrant glycosylation in cancer was first described more than 45 years ago [4], and since then it has been well documented that fundamental changes in the glycosylation patterns of cell surface and secreted glycoproteins occur during malignant transformation and cancer progression. Many of the first cancer-specific antibodies identified were directed against oncofetal antigens expressed on embryonic and tumour cells but not in adult tissues [5]. The importance of glycosylation in cancer is further emphasised by that fact that the majority of FDA-approved tumour markers are glycoproteins or glycan antigens [6–8]. The expression of cancer associated glycans such as sialyl-Lewis^X^ (SLe^X^), Thomsen-nouvelle antigen (Tn), and sialyl-Tn (sTn) antigen have been detected in virtually every cancer type [9].

Growing evidence supports crucial roles for glycosylation during all steps of tumour progression, and it is well established that glycans regulate tumour proliferation, invasion, metastasis and angiogenesis [10, 11]. Aberrant glycosylation is frequently cited as a hallmark of cancer [11–15], but is notably absent from both the original hallmarks paper [1] and from the next generation hallmarks [3]. The goal of this review is to highlight glycosylation as a mechanistic concept integral to the recognised hallmark traits. Unique to our discussion is our focus on how glycosylation enables acquisition of all the 10 currently accepted hallmarks of cancer cells.

## GLYCOSYLATION

Glycosylation is the enzymatic process that produces glycosidic linkages of saccharides to other saccharides, lipids or proteins [11]. Glycosylation is a frequent and well known post-translational protein modification, and probably much more frequent than phosphorylation. The glycome, or complete pattern of glycan modifications in a cell or tissue, is assembled by the synchronised action of numerous glycan modifying enzymes. These enzymes include glycosyltransferases and glycosidases that glycosylate various complex carbohydrates such as glycoproteins, glycolipids and proteoglycans. How much a given protein is glycosylated depends on the presence and frequency of glycosylation sites in the protein sequence, as well as the expression and activities of specific glycosylation enzymes within the cell or tissue [16].

The two most common mechanisms by which glycans are linked to proteins are O-linked glycosylation and N-linked glycosylation. In O-linked glycosylation, sugars are added incrementally to the hydroxyl oxygen of serine, threonine residues [17]. A common type of O-linked glycosylation is initiated *via* addition of GalNAc, which can then be extended into various different structures. Other types of O-glycans include those attached *via* O-mannose, and the β-N-acetylglucosamine (O-GlcNAc) [18–20]. In N-linked glycosylation preassembled blocks of 14 sugars are transferred co-translationally *via* the amide group of an asparagine residue and are then further processed [21]. Addition of O-GlcNAc (O-GlcNAcylation) occurs almost exclusively within the cell as an alternative to phosphorylation, while N- and O-glycans tend to be found at the cell surface as secreted entities, meaning that intra-cellular proteins may be effected by O-GlcNAcylation while interactions at the cell surface often involve N- glycans and O-glycans [17, 20, 22].

Alterations in glycan composition can aid in various stages of cancer progression. The mechanisms that produce altered glycan structures in cancer cells remain poorly understood, but are believed to involve changes in epigenetics, genetic mutations, misregulated expression of glycosyltransferase and chaperone genes, and mislocalisation of glycosyltransferases [23–26].

## SUSTAINING PROLIFERATIVE SIGNALLING

A fundamental trait of cancer cells is their ability to maintain chronic proliferation [1]. It is well established that glycan expression can play a role in maintaining proliferative signalling. O-GlcNAc modification of proteins has been shown to regulate important cell proteins involved in cell cycle progression including the transcription factor forkhead protein M1 (FoxM1), cyclin D1 [27], and cMYC [28]. Increased MYC O-GlcNAcylation can compete with phosphorylation, stabilising MYC protein and contributing to oncogenesis [28]. The degree of N-glycan branching can also modulate the activity and signalling of growth factor receptors, and can contribute to proliferative signalling [29–32]. Numerous growth factor receptors including EGFR, FGFR, PDGF, MET and IGFR are known to be regulated by glycosylation [33–35].

The extracellular matrix (ECM) imparts the spatial context for the signalling events of various cell surface growth factor receptors, and is composed of a dynamic and complex array of glycoproteins, collagens, glycosaminoglycans and proteoglycans [36]. Glycosylation has been shown to facilitate integrin dependent growth factor signalling to promote cell growth and survival [37, 38], and can also markedly modify the function and signalling of the multifunctional cell surface molecule CD44 [39, 40]. Ceramide glycosylation in the cell membrane can actively participate in maintaining cancer stem cells by activating c-Src signalling and β-catenin mediated upregulation of stem cell factors [41]. Proteoglycans also play a role in the biogenesis and recognition of exosomes (secreted vesicles of endosomal origin) which are involved in cell signalling [42].

## EVADING GROWTH SUPPRESSORS

In addition to inducing and maintaining positively acting growth stimulatory signals, cancer cells must also overcome powerful programs that negatively regulate cell proliferation, many of which depend on the actions of tumour suppressor genes. The two canonical suppressors of proliferation, p53 and RB (retinoblastoma) proteins have both been documented to contain potential glycosylation sites, and their functions may be controlled by dynamic O-GlcNAc modification as well as by phosphorylation [43–45]. O-GlcNAcylation of p53 at residue Ser149 is thought to promote its tumour suppressor activity by inhibiting its phosphorylation on Thr155 [44, 45]. Examples of gain of function p53 mutants have been widely described [46–48], and in this context it might be possible that O-GlcNAcylation induced stabilisation of gain of function mutant forms of p53 could amplify its pro-oncogenic activity [45].

## DEREGULATING CELLULAR ENERGETICS

A key feature of cancer cells is a shift from oxidative phosphorylation to aerobic glycolysis [49]. Known as the ‘Warburg effect’, this shift in metabolism is characterised by high rates of glucose and glutamine uptake to cope with the increased energetic and biosynthetic needs of the tumour. The abundance of glucose contributes to increased glycolysis and increased flux through metabolic pathways such as the hexosamine biosynthetic pathway (HBP). The end product of the HBP is UDP-GlcNAc which is a critical metabolite used in O-GlcNAcylation and in both N- and O-glycosylation [50]. O-GlcNAc is elevated in various types of cancer and has itself been described as a hallmark of cancer [45, 51]. O-GlcNAcylation can act as a ‘nutritional sensor’, and may provide feedback signals that modulate metabolism in response to changing nutrient status [20, 52, 53]. Several studies have suggested that hyper-O-GlcNAcylation is linked to cancer-associated metabolic reprogramming [54]. O-GlcNAc can modify a number of glycolytic enzymes [55–57], including phosphofructokinase 1 (PFK1) which catalyses the rate limiting step in glycolysis [57]. O-GlcNAcylation may also play a role in metabolic reprogramming by regulating transcription factors [58, 59] and c-MYC stability [28].

## RESISTING CELL DEATH

Programmed cell death by apoptosis serves as a natural mechanism to prevent cancer development, and a hallmark of cancer is the ability of malignant cells to evade apoptosis [1, 60]. Glycans play a key role in many of the processes leading to cell death, and can control intracellular signals and extracellular processes that promote the initiation, execution and resolution of apoptosis [61]. Cancer cells often use their glycosylation machinery to modify glycans on cell death receptors, enabling them to resist apoptosis [61]. Glycosylation can modulate the function of death receptors including Fas (CD95) and TNFR1 (tumour necrosis factor receptor 1) [62, 63]. The glycosylation of death receptors and their canonical ligands may critically regulate apoptosis by disrupting ligand-receptor interactions [64, 65], modulating the formation of signalling complexes [66], and influencing ligand secretion from effector cells [67]. The apoptotic machinery can be positively or negatively regulated through interactions between glycosylated receptors and glycan binding proteins [68]. Lectins are a family of carbohydrate binding proteins that specifically recognise glycans. Galectin-3 association with Fas can repress apoptotic signals [69], and increase tumour cell survival [70, 71].

Cellular accumulation of the glycosphingolipid GD3 contributes to mitochondrial damage and plays a key role in apoptosis [72]. GD3 expression is upregulated in neoplastic cells where it regulates tumour invasion and survival [73]. Although an increase in GD3 would normally induce apoptosis, in glioblastomas addition of an acetyl group to the terminal sialic acid (to produce 9-O-acetyl GD3) makes GD3 unable to induce apoptosis, thus promoting tumour survival [74]. Ceramide accumulation also plays a role in programmed cell death [75]. The glucosylceramide synthase (GCS) enzyme can glycosylate ceramide and blunt its pro-apoptotic activity in cancer cells [76].

## ENABLING REPLICATIVE IMMORTALITY

An essential property of cancer cells is to overcome the normal cellular senescence process resulting from the shortening of telomeres. Telomerase activation is a critical step in carcinogenesis and is thought to occur in over 90% of cancers [77]. Transcriptional reactivation of the human telomerase reverse transcriptase (hTERT) gene is a major mechanism of cancer-specific activation of telomerase. Although to date there is no evidence linking glycans to telomerase activation, and glycosylation of hTERT has so far not been reported, there is indirect evidence linking glycosylation to telomerase activation through the glycosylation of the transcription factor c-MYC. C-MYC is a direct mediator of telomerase activation and can directly induce hTERT gene expression [78, 79]. The c-MYC protein is known to be glycosylated [80], and has been shown to be stabilised by modification with O-GlcNAc [28]. Levels of O-GlcNAcylation are up-regulated in various types of cancer [45, 51], as are some of the enzymes involved in the hexosamine biosynthesis pathway [81]. Future studies will help determine whether O-GlcNAc mediated stabilisation of c-MYC can indirectly influence telomerase activation and contribute to replicative immortality.

## ACTIVATING INVASION AND METASTASIS

The development of malignant tumours requires the ability of tumour cells to overcome cell-cell adhesion and then invade surrounding tissue. Mounting evidence suggests that certain glycan structures can affect tumour cell invasiveness, including the ability to disseminate through the circulation and metastasise into distant organs [9]. Cancer cells often have high levels of sialylated glycans [82], which are often associated with malignancy and poor prognosis in patients [83–86]. Increased sialylation can increase local negative charges to physically disrupt cell-cell adhesion, and promote detachment from the tumour mass through electrostatic repulsion [87]. Consistent with this, expression of the cancer-associated sTn-antigen reduces cell adhesion in prostate cancer and increases migration and invasion in breast and gastric carcinoma [88–93]. Similarly, ectopic expression of the sialytransferase ST6GAL1 in breast cancer cells has been shown to reduce cell adhesion [94]. Cancer cells characteristically express proteins with truncated O-glycan structures that are thought to be due to mutations or epigenetic silencing of the COSMC gene [95, 96], or to increased expression of ST6GalNAc1 [88]. The immature O-glycophenotype of cancer cells has been directly linked to cancer cell growth and invasion [95].

Glycosylation can also influence the activity and localisation of proteins involved in cell adhesion, including the transmembrane glycoprotein E-cadherin. Over-expression of the enzyme MGAT5 in gastric cancer cells induces E-cadherin mislocalisation from the cell membrane into the cytoplasm [97, 98]. MGAT5 catalyses β1,6GlcNAc branching of N-glycans on E-cadherin, which in turn leads to non-functional adherens junctions, impairs cell-cell adhesion and downstream signalling, and contributes to invasion and metastasis [22, 97–101]. Downregulation of the enzyme MGAT3 in mouse mammary tumours increases cell migration and metastasis but genetic background may modify this effect in human breast cancer cells [22, 102, 103]. MGAT3 catalyses the addition of bisecting GlcNAc to complex N-glycans and is thought to influence interactions with galectins, and to regulate the function of some glycoproteins, including growth factor receptors and some adhesion molecules [22].

As well as reducing cell-cell adhesion and aiding dissociation from the primary tumour, glycans can also promote the adhesion of tumour cells. The SLe^X^ antigen is upregulated in several types of cancer [17, 104], and can promote adhesion of tumour cells to endothelial cells through interactions with selectins, in this way mediating the initial steps in metastasis [11, 82]. Galectin-3 regulates the dynamics of N-cadherin [29], and Galectin-1 binding to CD44 and CD326 can promote attachment to the ECM and endothelial cells [105].

The sialyltransferase ST6GalNAc2 has been identified as a metastasis suppressor in breast cancer cells which is linked to patient survival [106]. Loss of ST6GalNAc2 was found to alter the profile of O-glycans on the cell surface and facilitate Galectin-3 binding, leading to an increased metastatic burden [106]. Glycosylation enzymes may also play a key role in mediating cancer cell passage through the blood brain barrier. GALNT9 (an initiator of O-glycosylation) is frequently epigenetically dysregulated in breast tumours that metastasise to the brain [107]. The sialyltransferase ST6GalNAc5 is normally restricted to the brain, but its expression in breast cancer can specifically mediate metastasis to the brain, highlighting the role of cell-surface glycosylation in organ-specific metastatic interactions [108].

## INDUCING ANGIOGENESIS

Through inducing the process of angiogenesis, development of tumour associated neovasculature enables tumours to acquire nutrients and oxygen as well as the ability to remove metabolic waste including carbon dioxide. The development of vasculature involves growing new endothelial cells and their assembly into tubes (vasculogenesis), and the sprouting (angiogenesis) of new vessels from existing ones. In the adult the vasculature is largely quiescent, but during tumour progression an ‘angiogenic switch’ is activated causing vasculature to continually sprout new vessels and aid tumour growth [109]. A distinct set of glycosylation related genes has been linked to the angiogenesis process [110, 111], and it has become increasingly evident that glycans are integral to different events in the angiogenesis cascade [112].

A key inducer of angiogenesis is vascular endothelial growth factor (VEGF), which signals *via* receptor tyrosine kinases (VEGFRs) and plays a pivotal role in angiogenesis during development and in cancer. Glycosylation of both VEGF and the VEGFRs is associated with angiogenesis. VEGF levels are upregulated by O-GlcNAcylation [113], and aberrant glycosylation of VEGFR can modulate its interaction with galectins and influence blood vessel growth [112]. Glycans also play a role in angiogenesis by regulating Notch signalling [114], maintaining endothelial cell survival [115], controlling vascular permeability [116], and mediating the connection of blood and lymphatic vessels [117]. Changes in cytokines, growth factors and hypoxic conditions have been shown to alter the endothelial glycome to facilitate binding of galectin-1 and activate pro-angiogenic signalling pathways, raising the possibility that a glycosylation signature could be used to distinguish blood vessels at different stages of tumour progression [118].

Heparan sulfate (HS) proteoglycans are abundantly expressed in the developing and mature vasculature, and play a pivotal role in angiogenesis by facilitating the binding of cell surface pro-angiogenic growth factors [119–121]. HS proteoglycans have been described as ‘heavy hitters in the angiogenesis arena’ [122], and can modulate angiogenesis by affecting the bioavailability and interaction of heparin-binding VEGFs with VEGFRs [123, 124], and by interacting with anti-angiogenic factors such as endostatin [125]. In ovarian cancer HS has been shown to impact angiogenesis through EGF receptor signalling and influencing the expression of angiogenic cytokines [126].

## GENOME INSTABILITY & MUTATION

Acquisition of the cancer hallmarks is made possible in part by the development of genomic instability in cancer cells which generates random mutations and chromosomal rearrangements. The accumulation of mutations can be accelerated by disrupting the surveillance systems that normally monitor genomic integrity. The tumor suppressor p53 has long been known to play a central role in maintaining a stable genome [127]. O-GlcNAc and O-phosphate modifications co-ordinately regulate p53 stability and activity [44], and a role for O-GlcNAc in the regulation of DNA damage signalling or repair has been suggested [128]. ATM, a key regulator of DNA damage repair is glycosylated, and studies have indicated a dynamic interplay between phosphorylation and O-GlcNAc in the regulation of the DNA damage pathway which could be linked to genomic instability in cancer [129].

## TUMOUR PROMOTING INFLAMMATION

It has long been recognised that some tumours are densely infiltrated by cells of the immune system and thereby mirror inflammatory conditions in non-neoplastic tissues [3, 130]. Historically, these immune responses were thought to reflect an attempt by the immune system to eradicate the cancerous cells, but there is now growing evidence that the response has an unanticipated paradoxical effect to actually aid in tumorigenesis and cancer progression. Within the tumour microenvironment, inflammation can contribute to multiple hallmark capabilities [2, 3], and plays a role in the proliferation and survival of malignant cells, angiogenesis, metastasis, subversion of adaptive immunity, and response to hormones and chemotherapy [2, 131–134]. Genomic instability can also be induced by inflammatory mediators [2]. Changes in glycan composition are closely associated with inflammation [14], and suggest an intricate relationship between glycosylation and inflammation in cancer progression. The selectin proteins (E-, P- and L-Selectin) are associated with cancer metastasis [135], but also play a key role in the entry of circulating lymphocytes into peripheral lymph nodes and leukocyte emigration into inflamed tissues [14, 136]. The selectins bind sialylated and fucosylated glycans (such as SLe^x^) which act as ‘endothelial zip codes’ for the homing of lymphocytes into inflammatory sites [137].

Emerging evidence suggests that key mediators in the inflammatory response may be regulated by glycosylation. NF-κB is a well-characterised orchestrator of inflammation which induces the expression of inflammatory cytokines [138]. The transcriptional activity of NF-κB can be regulated by O-GlcNAcylation [139], which is known to be upregulated in multiple cancer types [45]. Similarly, the pro-inflammatory molecule COX2 is also regulated by glycosylation [140], and the efficiency of some COX2 inhibitors is thought to be dependent on COX2 glycosylation state [141]. Interestingly, a diet derived sialic acid called N-glycolylneuraminic acid (Neu5Gc, found primarily in red meat) can be incorporated in human tissues. This can lead to the production of auto-antibodies against Neu5Gc and subsequent tumour related inflammation *via* induction of ‘xenosialitis’[142].

As well as glycan involvement in the inflammatory response, the inflammatory microenvironment can also reciprocally mediate changes in the glycan composition of cells, which could contribute to tumour malignancy. Pro-inflammatory cytokines can increase the expression of glycosyltransferases involved in the biosynthesis of cancer-associated antigens in pancreatic and gastric cancer cell lines [143, 144].

## AVOIDING IMMUNE DESTRUCTION

Cancer immune surveillance is thought to inhibit carcinogenesis and is an important host protection process through which transformed cells are eliminated by immune effector cells. Growing evidence suggests that interactions between tumour specific glycans and lectins on immune cells are involved in modulating the tumour microenvironment [145]. Glycans regulate various aspects of the immune response and can interfere with the anti-tumour response of the immune system, leading to the emergence of cancer cells resistant to the immune system [11, 146]. This process is mediated by various lectins that bind glycans and regulate immune processes [147, 148]. Galectins can modulate the immune and inflammatory responses and are thought to play a role in helping tumours escape immune surveillance [147, 149]. Siglecs (sialic acid-binding immunoglobulin-type lectins) are transmembrane proteins found on the surface of immune cells. Siglecs are thought to bind to specific glycans and may play a role in escaping immune surveillance in cancer [150]. For example, the alteration of cell surface glycans can modulate siglec-7 mediated cytotoxicity of NK cells and contribute to immune evasion [151]. Glycosylation of IgG is known to play a role in tumour immune surveillance and is being investigated as a diagnostic marker in several cancer types [152–155]. Targeting altered glycosylation using anticancer vaccines that target tumour associated antigens is an appealing option for cancer treatment [156, 157].

## CONCLUSIONS

**Figure 1:**
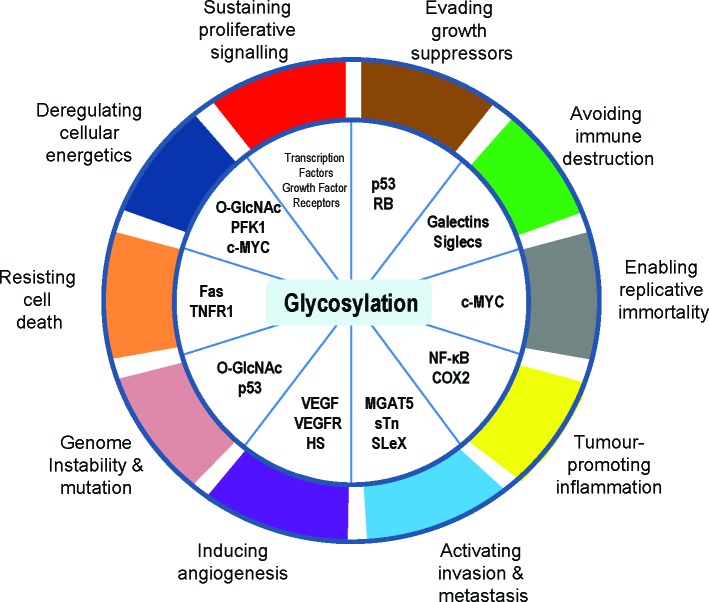
Glycosylation is an enabling characteristic that is causally associated with the acquisition of all the cancer hallmark capabilities

The hallmarks of cancer comprise biological capabilities acquired during the multi-step development of cancer that allow cancer cells to survive, proliferate and disseminate [1]. Glycosylation is frequently cited as hallmark of cancer but was notably absent from both the original hallmarks and from the updated next generation cancer hallmarks. Here we argue that the process of glycosylation is an enabling characteristic that is causally associated with the acquisition of all the proposed cancer hallmark capabilities (Figure 1), and conversely that the glycan composition of cancer cells can in turn be influenced by the other hallmarks. Glycans have roles in cancer cell signalling, tumour cell dissociation and invasion, cell-matrix interactions, angiogenesis, metastasis and immune modulation. Recognition of the widespread applicability of glycosylation to the cancer hallmarks will increasingly affect the development of new means to treat human cancer.
